# Supplementary figures and images for: Head-to-head comparison of three experimental methods of quantifying competitive fitness in C. elegans
Source: PLoS One. 2018 Oct 19;13(10):e0201507. doi: 10.1371/journal.pone.0201507 (PMC6195253; doi:10.1371/journal.pone.0201507)

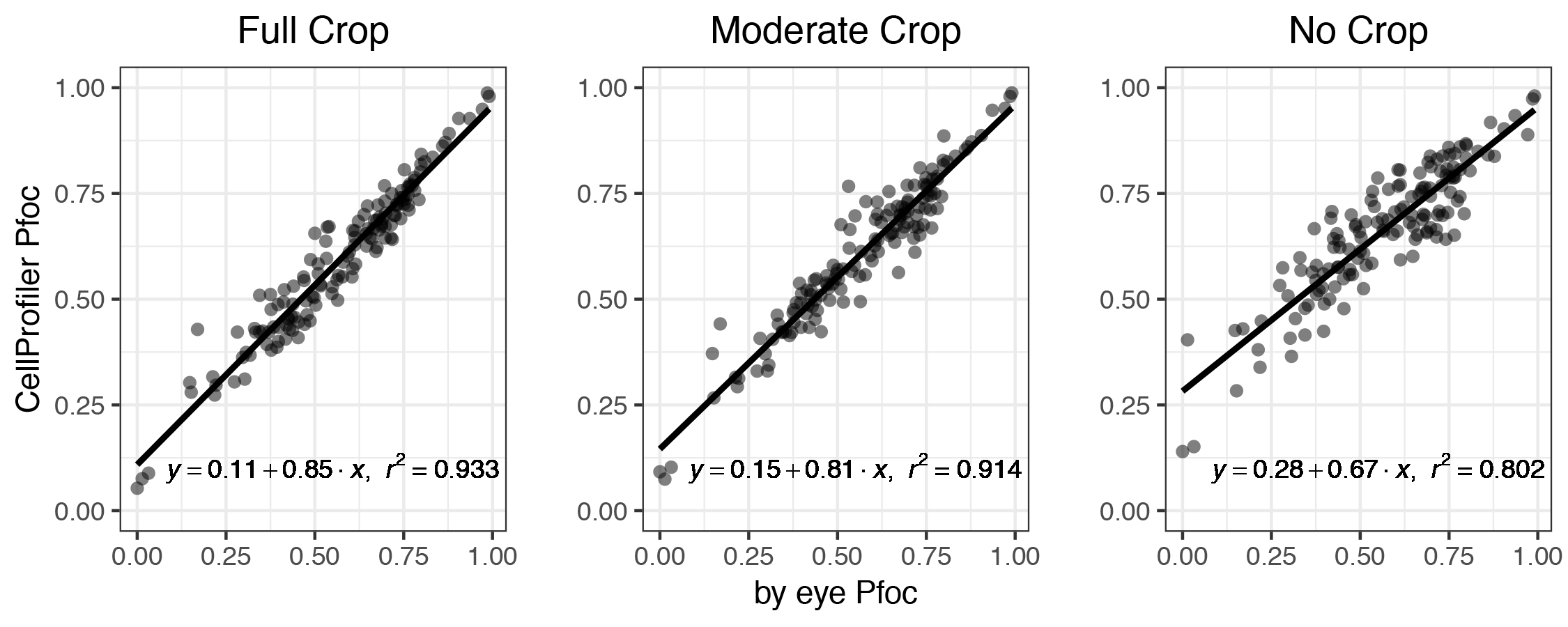

Supplement: S1 Fig — The proportion of wild-type worms (pfoc) estimated by CellProfiler is regressed against the same proportion estimated from the by eye count from the full crop of the same image. See methods for description of the cropping protocol. (TIF) [file pone.0201507.s001.tif]

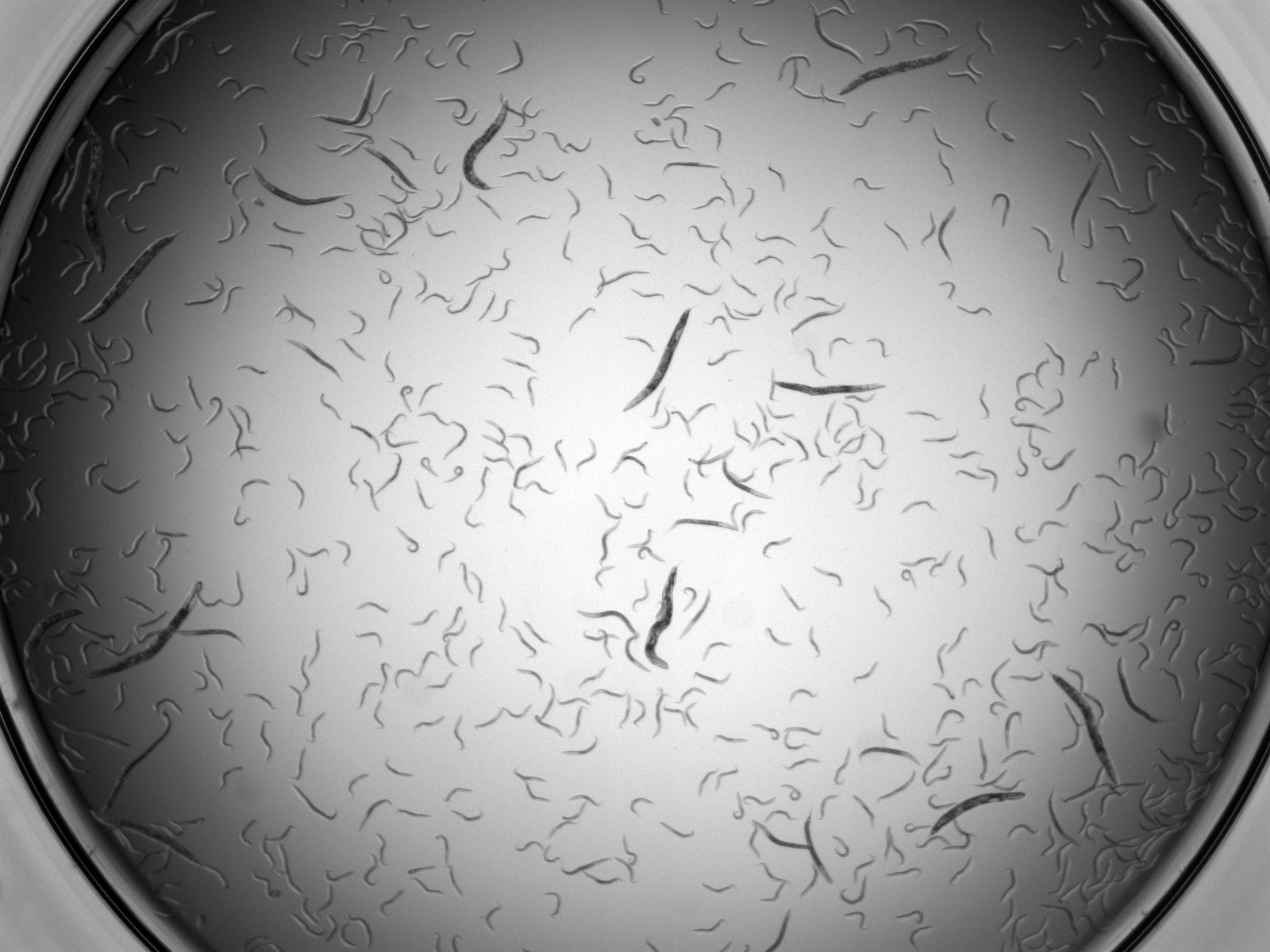

Supplement: S1 File — CellProfiler project files 1–2 are used to train and create worm models. CellProfiler project file 3 is used to score focal and competitor worms in image pairs and output scores as .csv files. (ZIP) [file pone.0201507.s005.zip › File S3/Competitive_Fitness_Images/Assay 12 BF/BF_12_1_A01.tif]

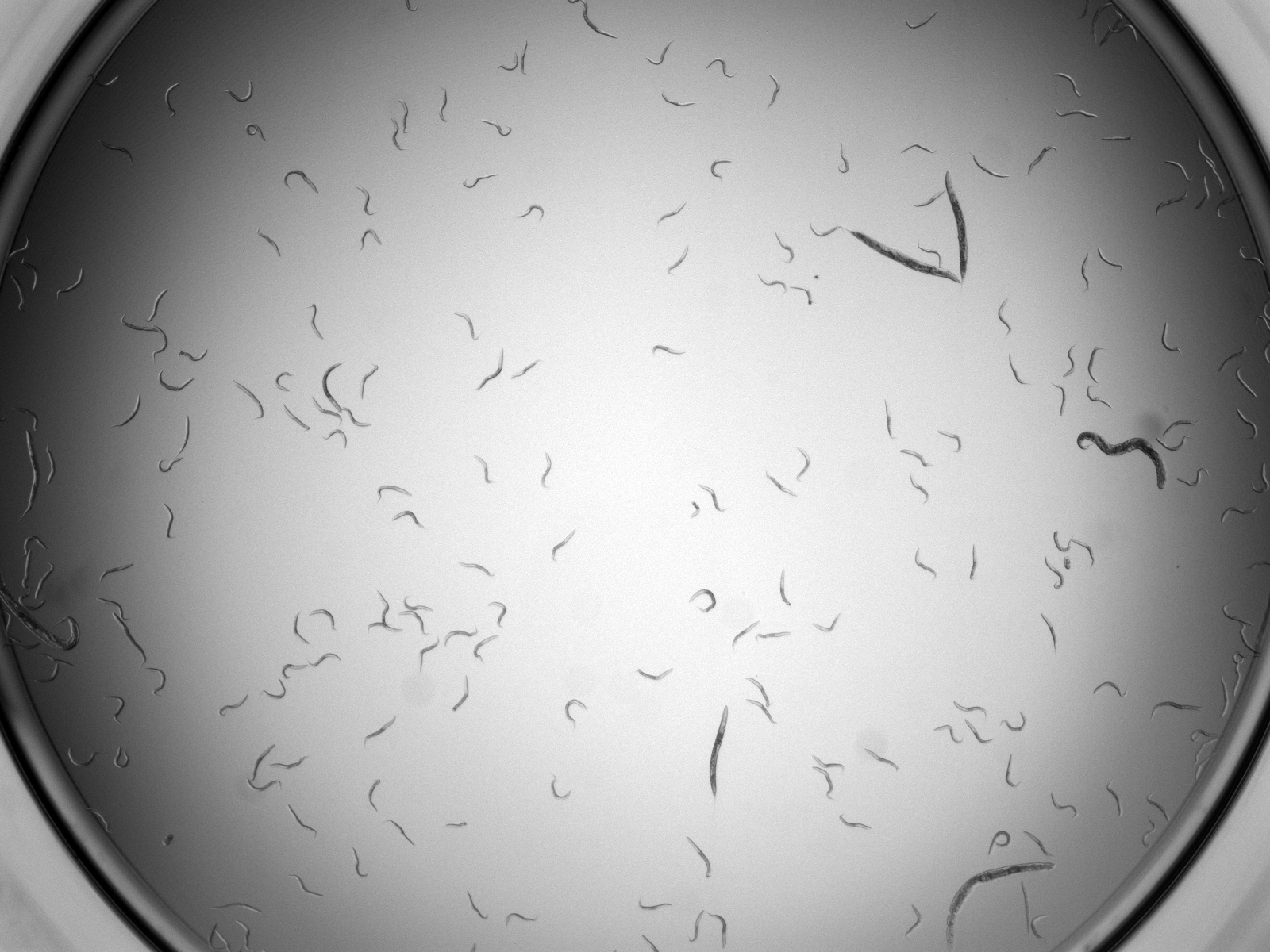

Supplement: S1 File — CellProfiler project files 1–2 are used to train and create worm models. CellProfiler project file 3 is used to score focal and competitor worms in image pairs and output scores as .csv files. (ZIP) [file pone.0201507.s005.zip › File S3/Competitive_Fitness_Images/Assay 12 BF/BF_12_1_A06.tif]

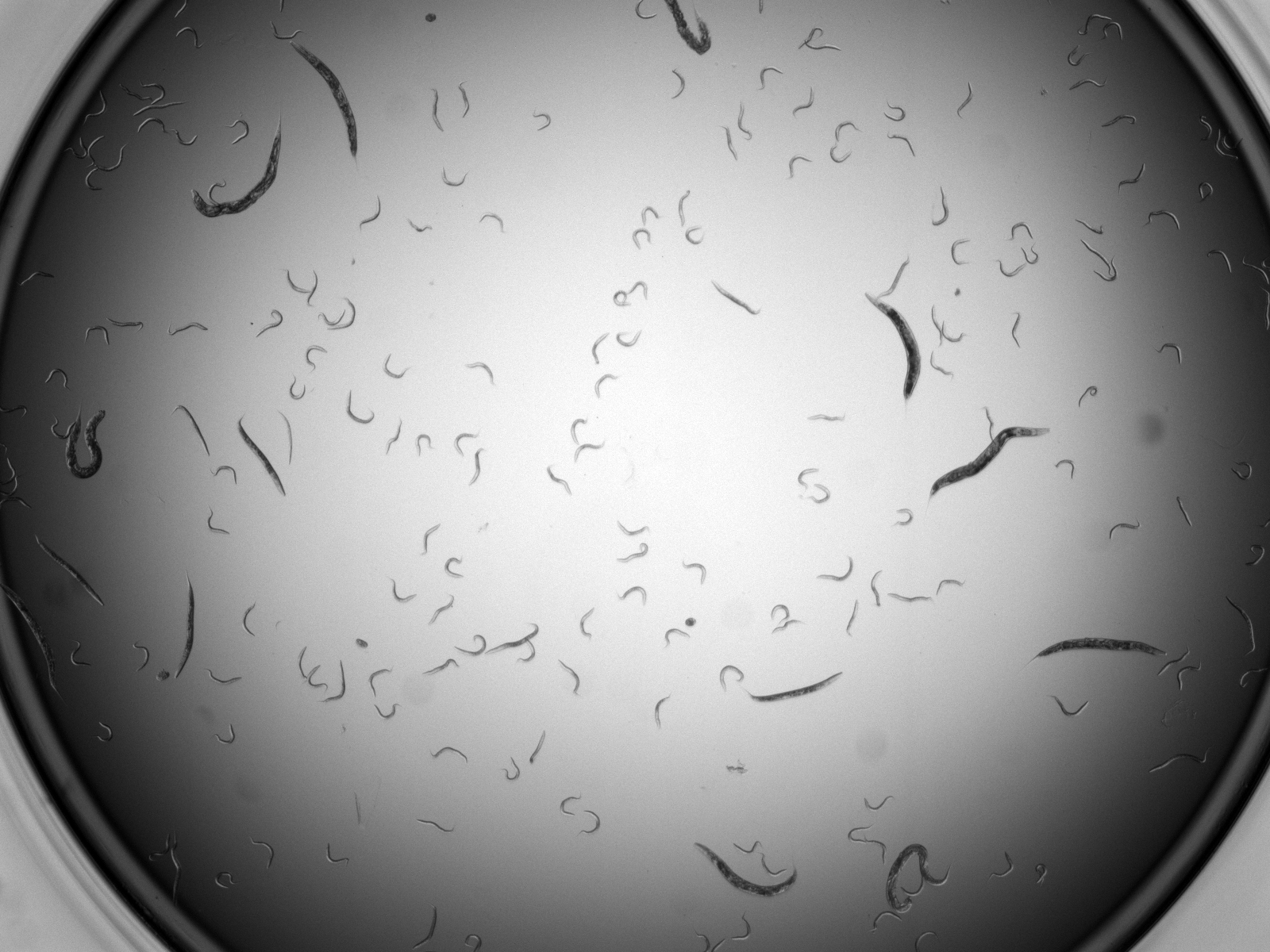

Supplement: S1 File — CellProfiler project files 1–2 are used to train and create worm models. CellProfiler project file 3 is used to score focal and competitor worms in image pairs and output scores as .csv files. (ZIP) [file pone.0201507.s005.zip › File S3/Competitive_Fitness_Images/Assay 12 BF/BF_12_1_C05.tif]

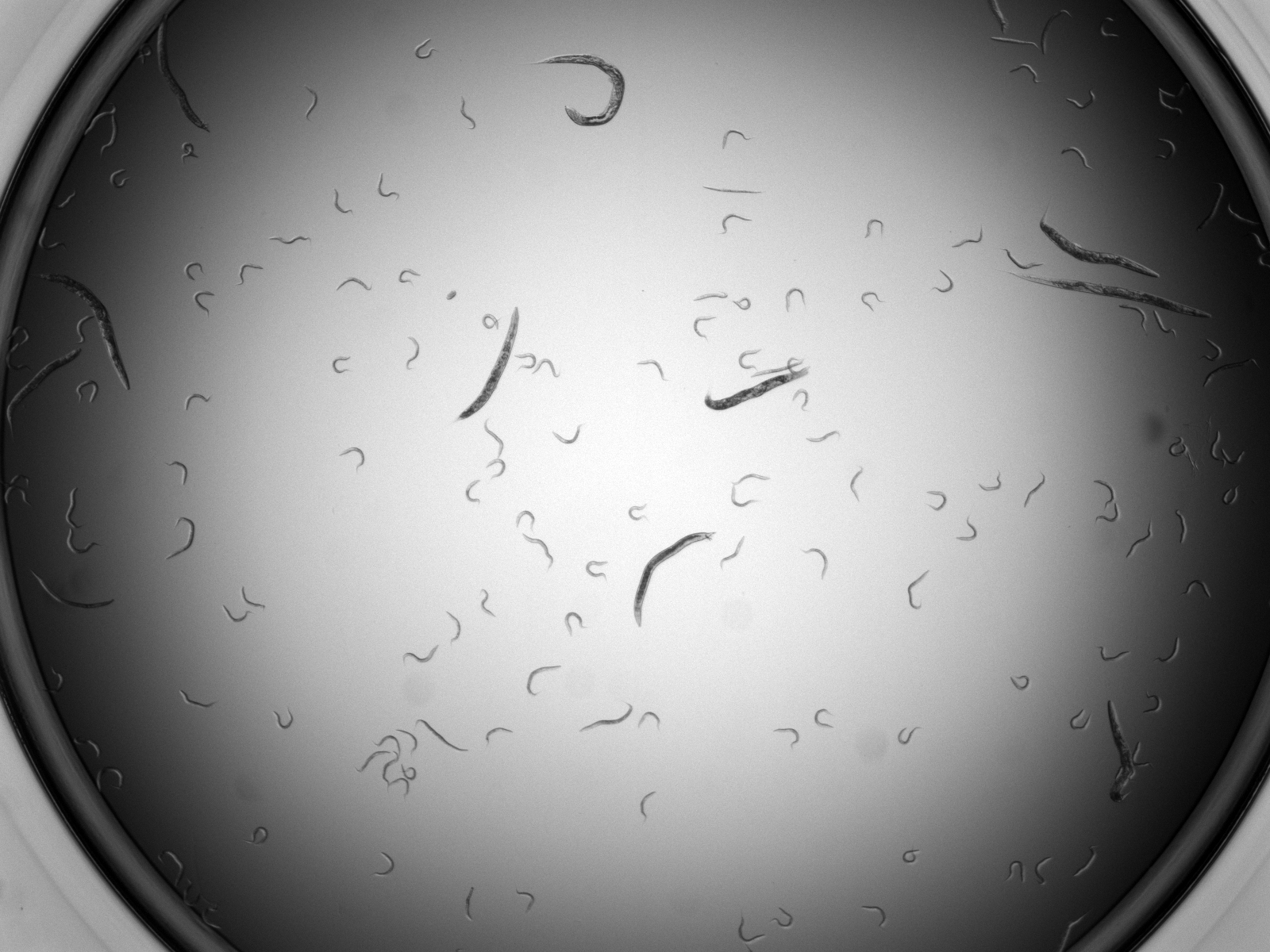

Supplement: S1 File — CellProfiler project files 1–2 are used to train and create worm models. CellProfiler project file 3 is used to score focal and competitor worms in image pairs and output scores as .csv files. (ZIP) [file pone.0201507.s005.zip › File S3/Competitive_Fitness_Images/Assay 12 BF/BF_12_1_D02.tif]

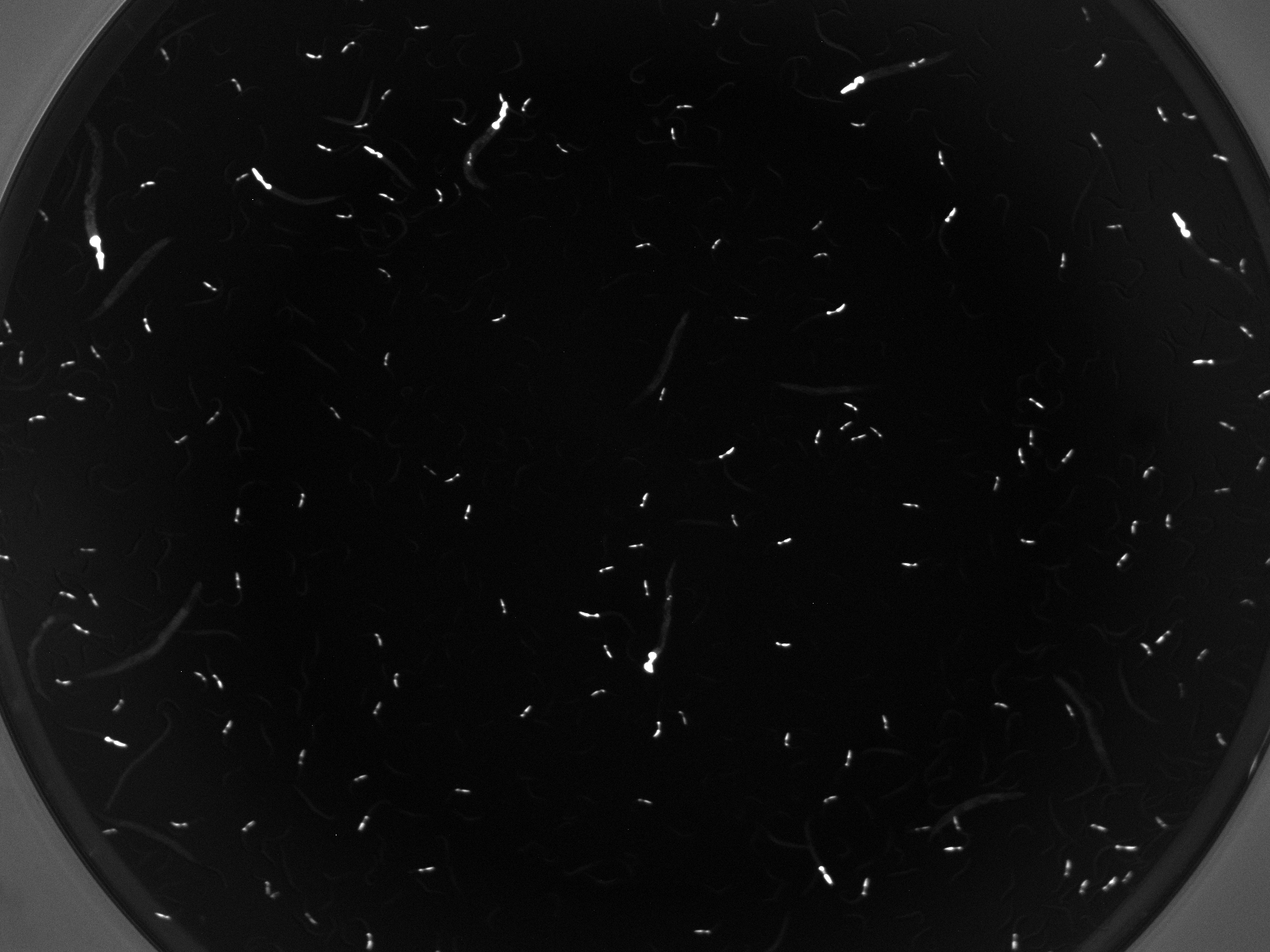

Supplement: S1 File — CellProfiler project files 1–2 are used to train and create worm models. CellProfiler project file 3 is used to score focal and competitor worms in image pairs and output scores as .csv files. (ZIP) [file pone.0201507.s005.zip › File S3/Competitive_Fitness_Images/Assay 12 GFP/GF_12_1_A01.tif]

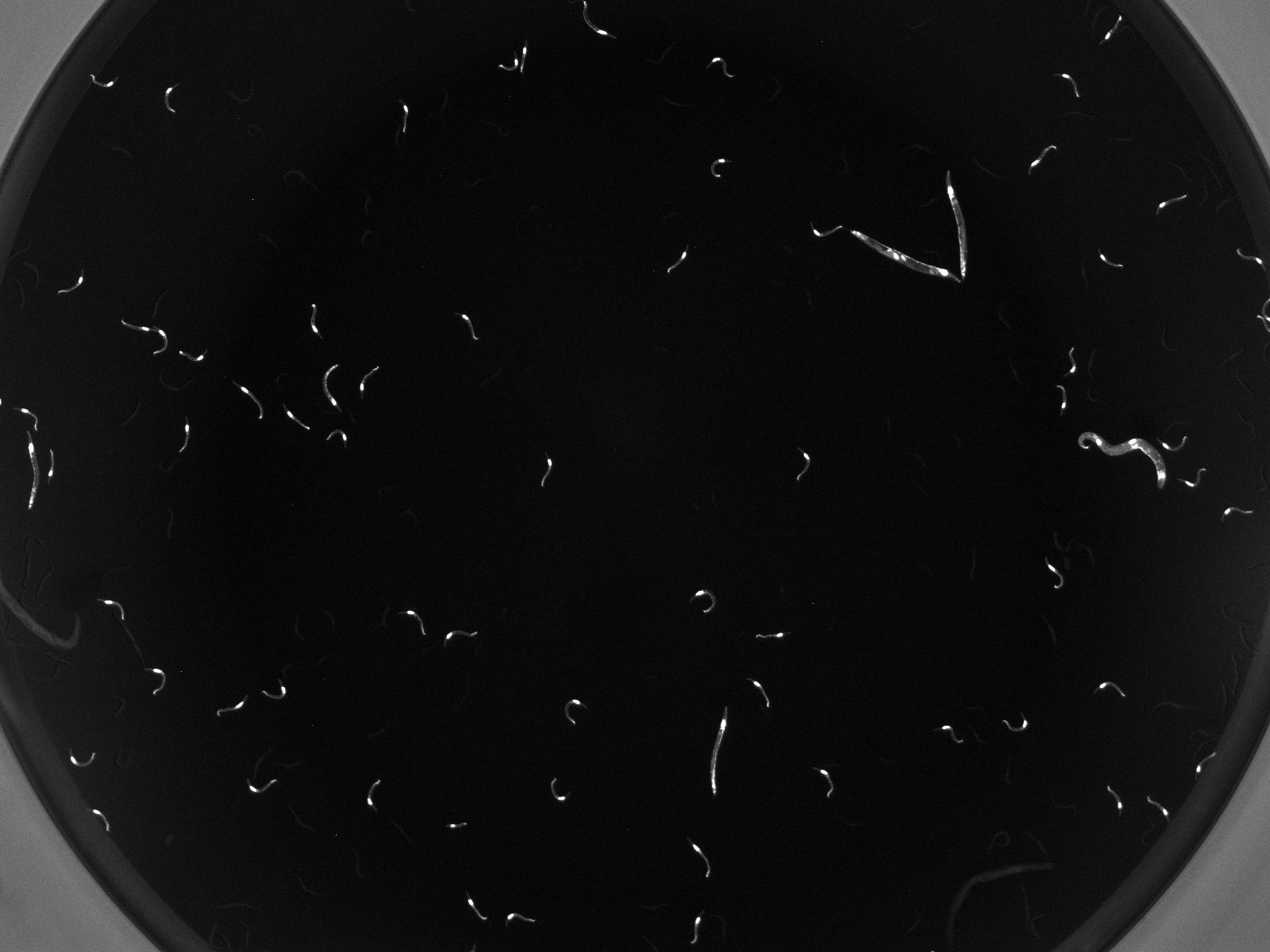

Supplement: S1 File — CellProfiler project files 1–2 are used to train and create worm models. CellProfiler project file 3 is used to score focal and competitor worms in image pairs and output scores as .csv files. (ZIP) [file pone.0201507.s005.zip › File S3/Competitive_Fitness_Images/Assay 12 GFP/GF_12_1_A06.tif]

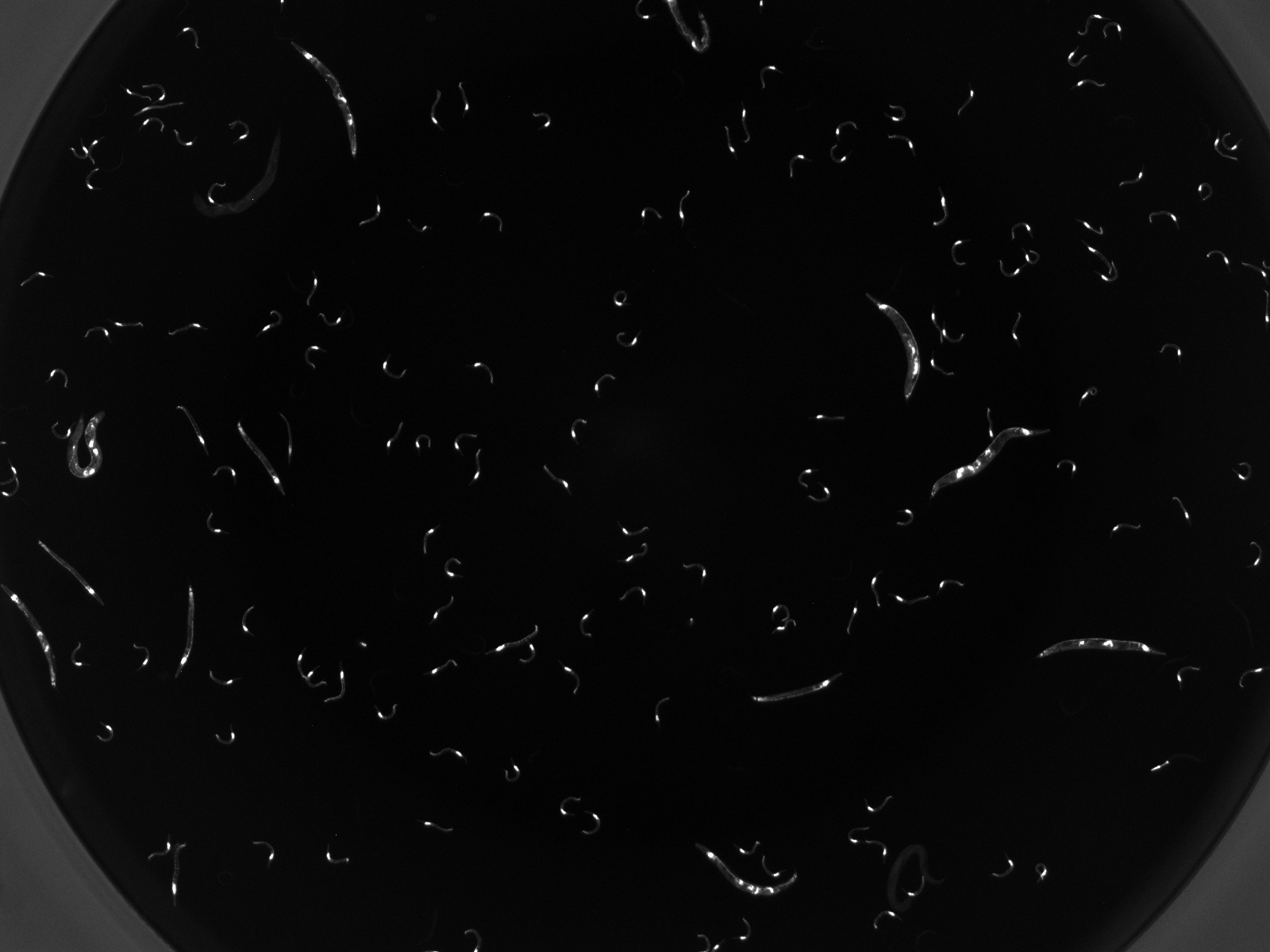

Supplement: S1 File — CellProfiler project files 1–2 are used to train and create worm models. CellProfiler project file 3 is used to score focal and competitor worms in image pairs and output scores as .csv files. (ZIP) [file pone.0201507.s005.zip › File S3/Competitive_Fitness_Images/Assay 12 GFP/GF_12_1_C05.tif]

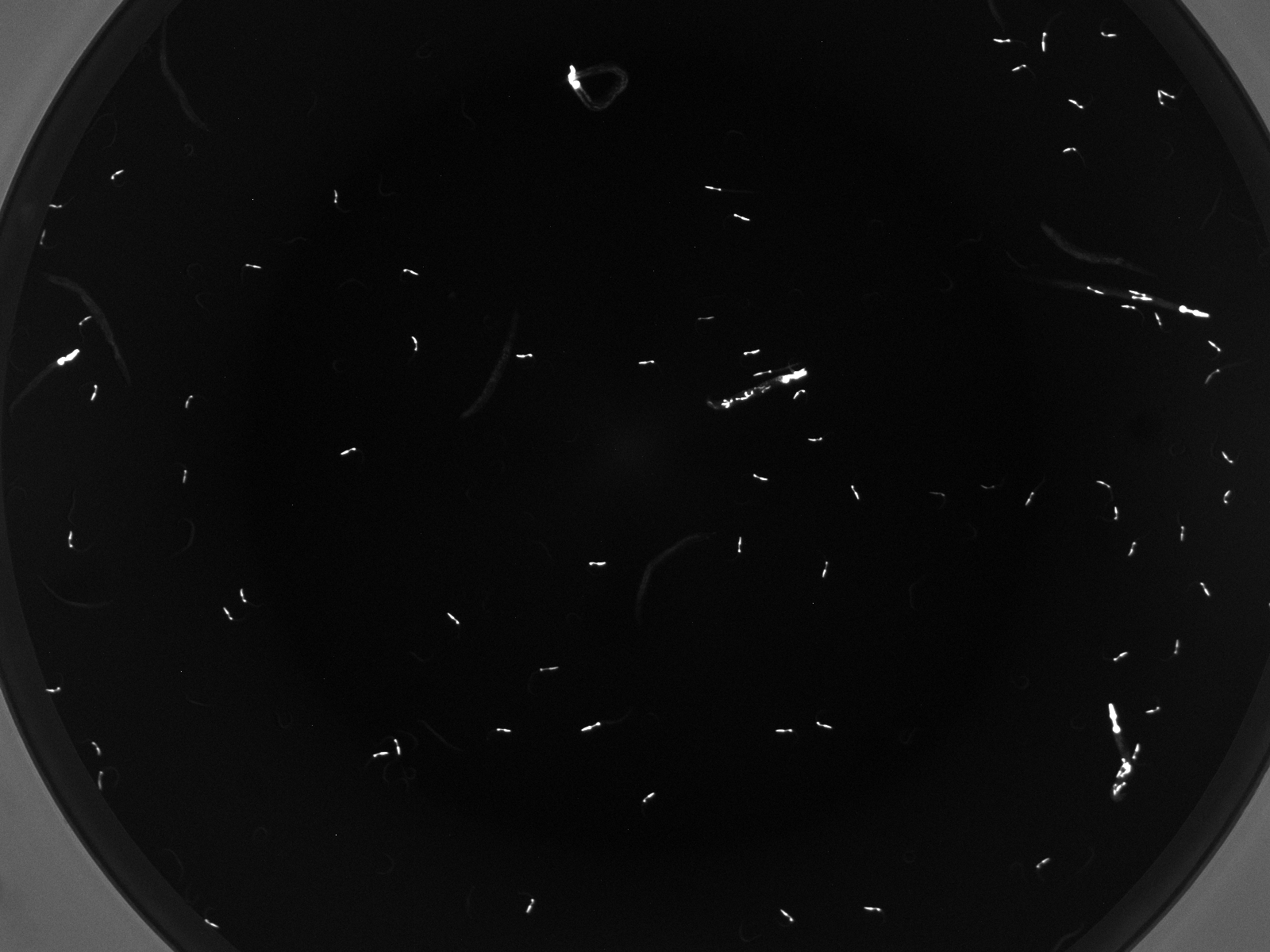

Supplement: S1 File — CellProfiler project files 1–2 are used to train and create worm models. CellProfiler project file 3 is used to score focal and competitor worms in image pairs and output scores as .csv files. (ZIP) [file pone.0201507.s005.zip › File S3/Competitive_Fitness_Images/Assay 12 GFP/GF_12_1_D02.tif]

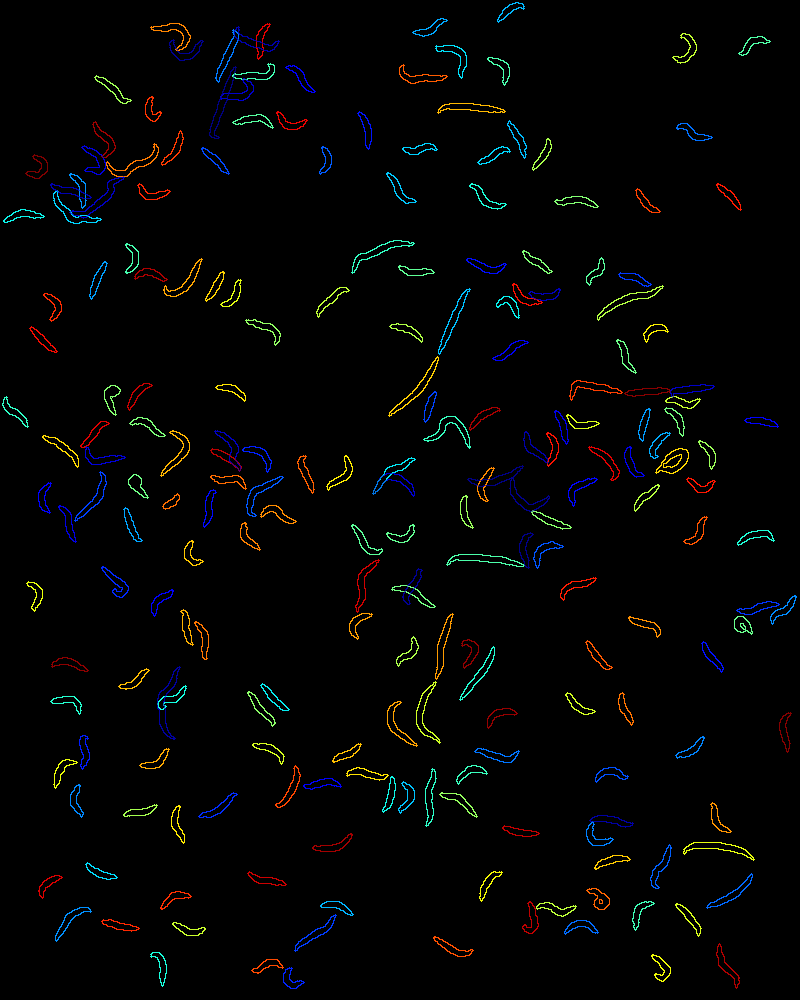

Supplement: S1 File — CellProfiler project files 1–2 are used to train and create worm models. CellProfiler project file 3 is used to score focal and competitor worms in image pairs and output scores as .csv files. (ZIP) [file pone.0201507.s005.zip › File S3/CP_outputs/Assay_12_output/BF_12_1_A01_outlines.png]

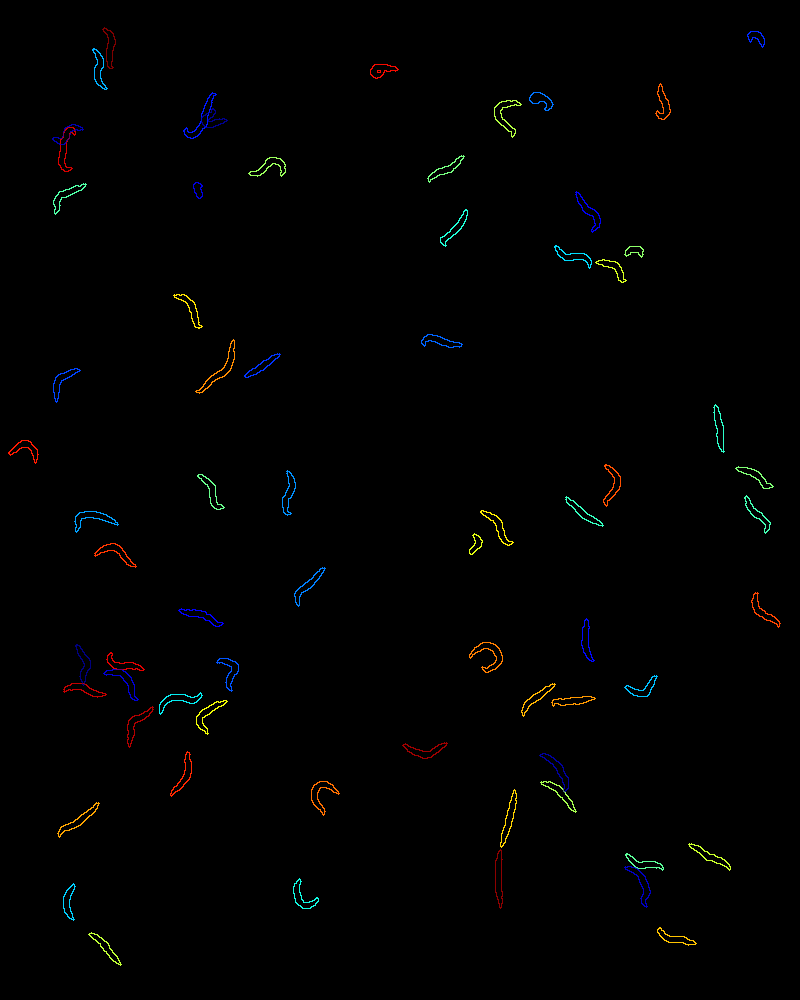

Supplement: S1 File — CellProfiler project files 1–2 are used to train and create worm models. CellProfiler project file 3 is used to score focal and competitor worms in image pairs and output scores as .csv files. (ZIP) [file pone.0201507.s005.zip › File S3/CP_outputs/Assay_12_output/BF_12_1_A06_outlines.png]

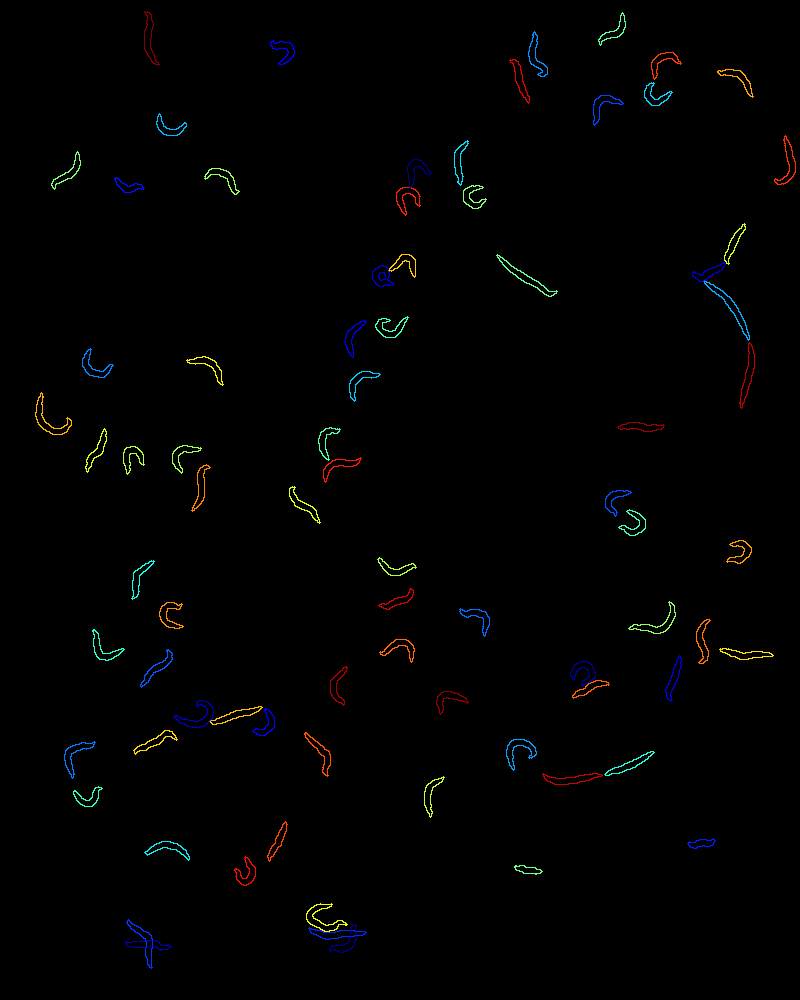

Supplement: S1 File — CellProfiler project files 1–2 are used to train and create worm models. CellProfiler project file 3 is used to score focal and competitor worms in image pairs and output scores as .csv files. (ZIP) [file pone.0201507.s005.zip › File S3/CP_outputs/Assay_12_output/BF_12_1_C05_outlines.png]

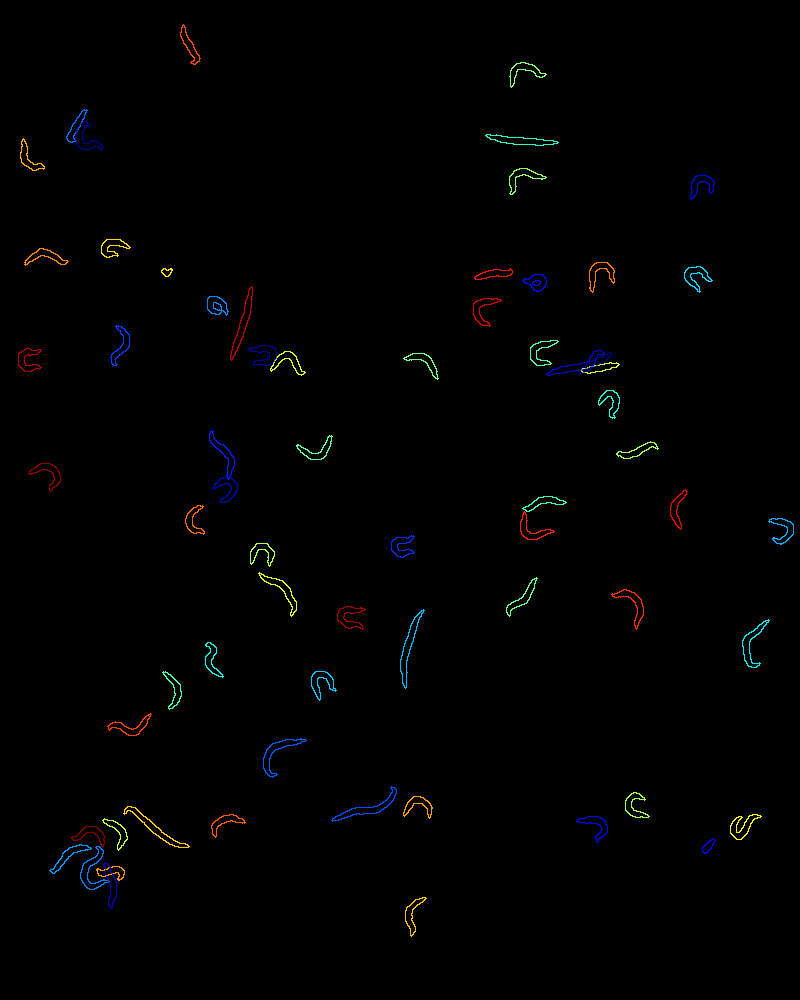

Supplement: S1 File — CellProfiler project files 1–2 are used to train and create worm models. CellProfiler project file 3 is used to score focal and competitor worms in image pairs and output scores as .csv files. (ZIP) [file pone.0201507.s005.zip › File S3/CP_outputs/Assay_12_output/BF_12_1_D02_outlines.png]

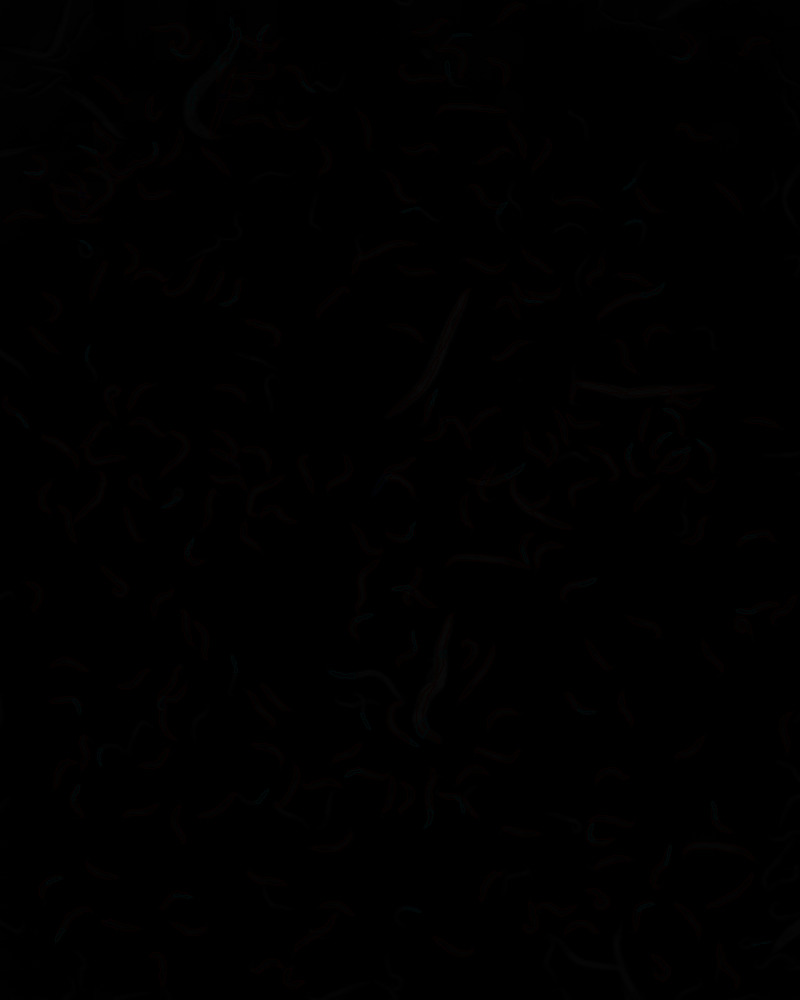

Supplement: S1 File — CellProfiler project files 1–2 are used to train and create worm models. CellProfiler project file 3 is used to score focal and competitor worms in image pairs and output scores as .csv files. (ZIP) [file pone.0201507.s005.zip › File S3/CP_outputs/Assay_12_output/GF_12_1_A01_GFP.png]

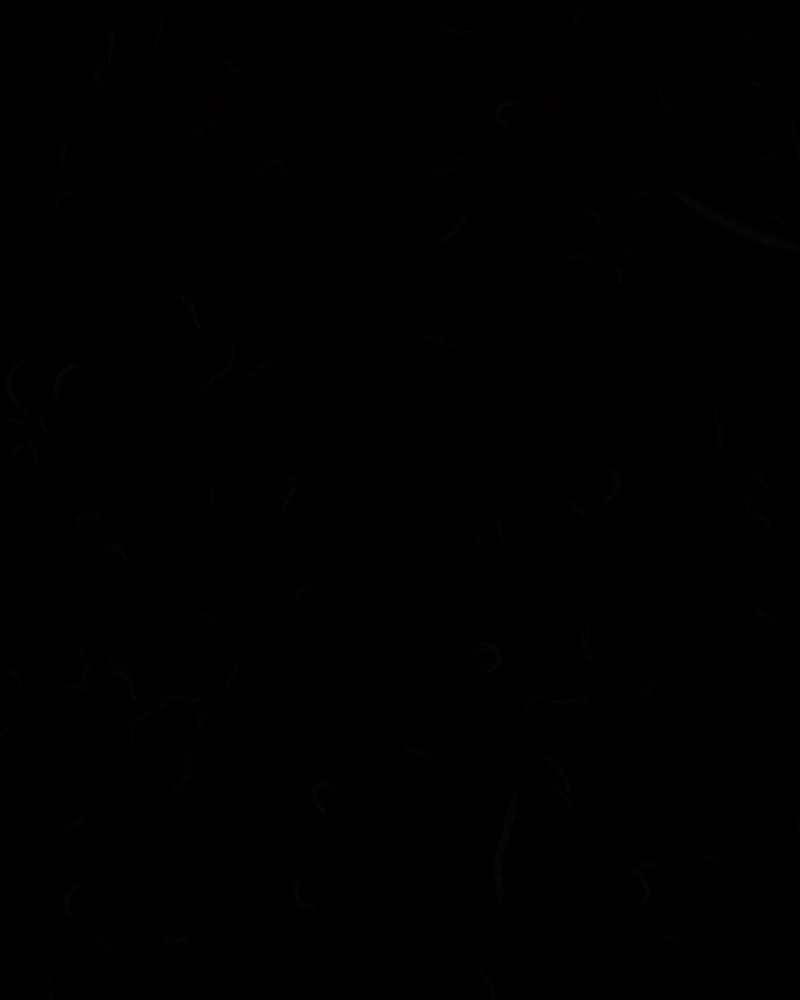

Supplement: S1 File — CellProfiler project files 1–2 are used to train and create worm models. CellProfiler project file 3 is used to score focal and competitor worms in image pairs and output scores as .csv files. (ZIP) [file pone.0201507.s005.zip › File S3/CP_outputs/Assay_12_output/GF_12_1_A06_GFP.png]

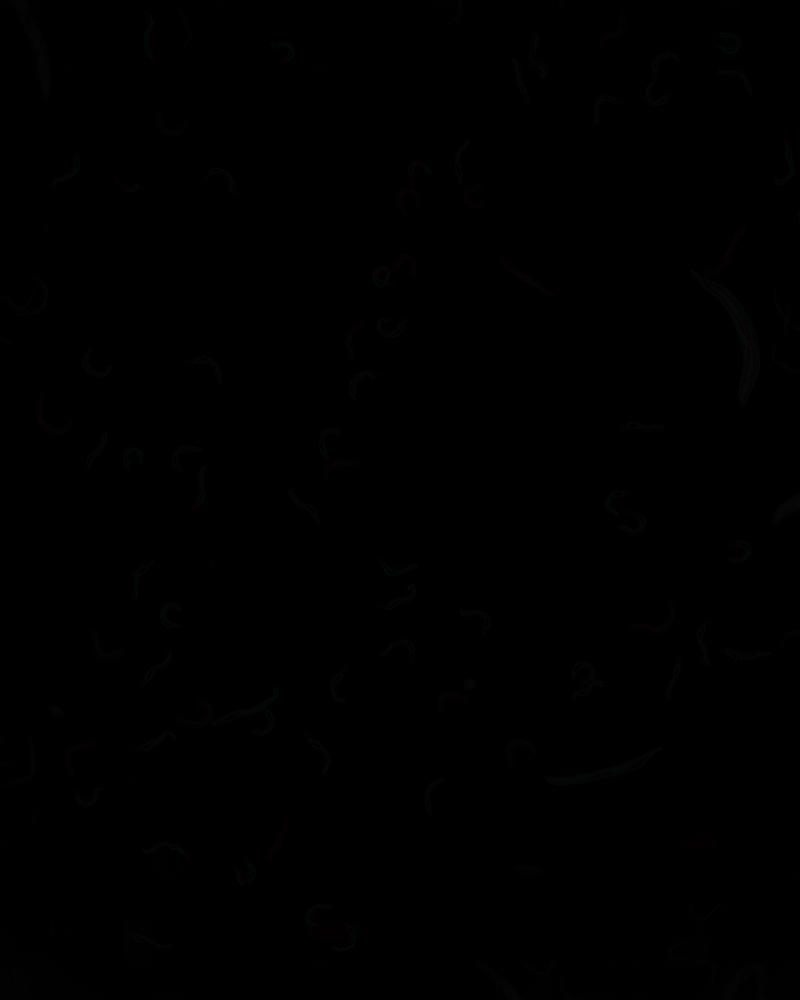

Supplement: S1 File — CellProfiler project files 1–2 are used to train and create worm models. CellProfiler project file 3 is used to score focal and competitor worms in image pairs and output scores as .csv files. (ZIP) [file pone.0201507.s005.zip › File S3/CP_outputs/Assay_12_output/GF_12_1_C05_GFP.png]

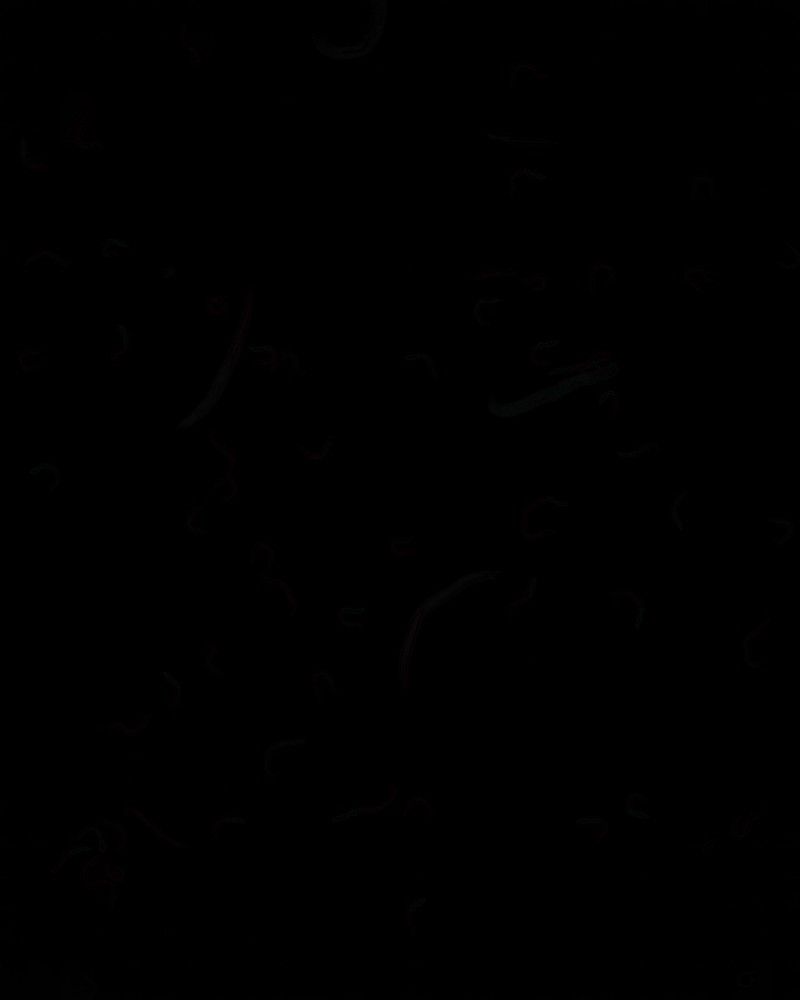

Supplement: S1 File — CellProfiler project files 1–2 are used to train and create worm models. CellProfiler project file 3 is used to score focal and competitor worms in image pairs and output scores as .csv files. (ZIP) [file pone.0201507.s005.zip › File S3/CP_outputs/Assay_12_output/GF_12_1_D02_GFP.png]

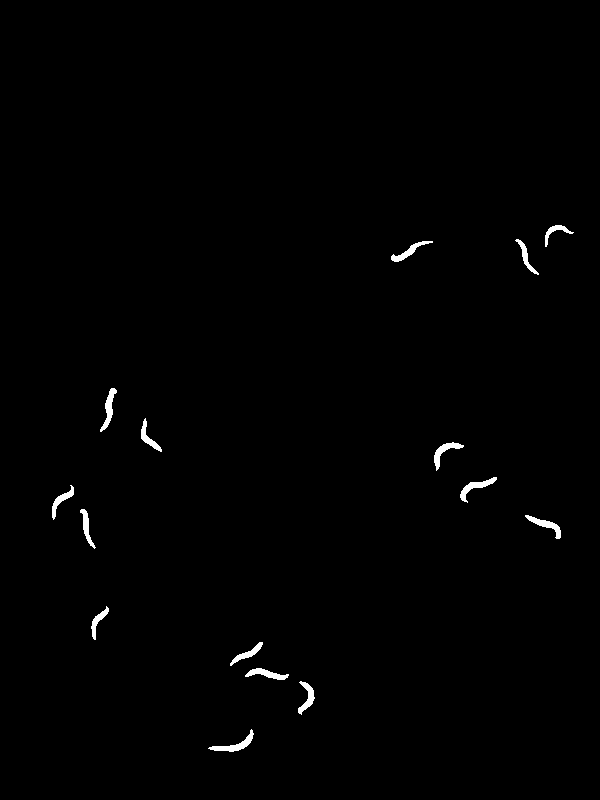

Supplement: S1 File — CellProfiler project files 1–2 are used to train and create worm models. CellProfiler project file 3 is used to score focal and competitor worms in image pairs and output scores as .csv files. (ZIP) [file pone.0201507.s005.zip › File S3/CP_outputs/worm_model_training_output/Assay_12_worm_model_trianing_output/BF_12_1_A01_SingleWorms.png]

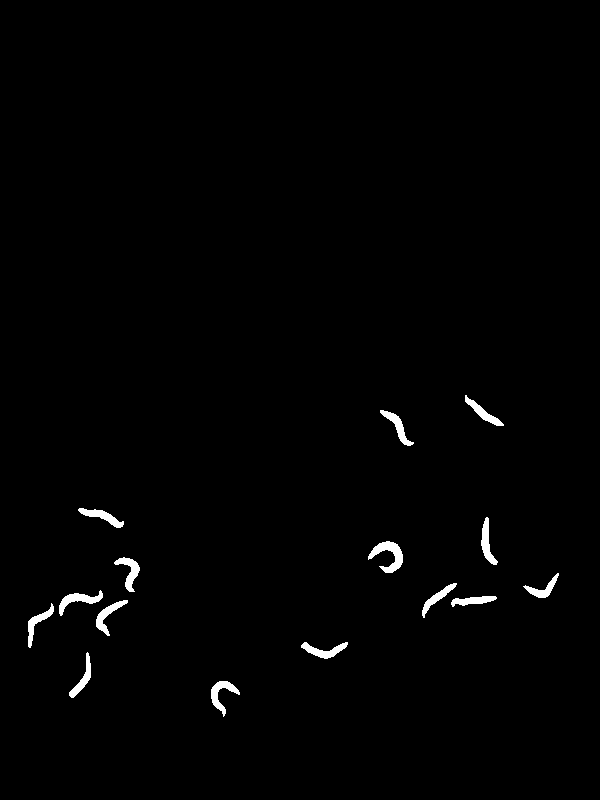

Supplement: S1 File — CellProfiler project files 1–2 are used to train and create worm models. CellProfiler project file 3 is used to score focal and competitor worms in image pairs and output scores as .csv files. (ZIP) [file pone.0201507.s005.zip › File S3/CP_outputs/worm_model_training_output/Assay_12_worm_model_trianing_output/BF_12_1_A06_SingleWorms.png]

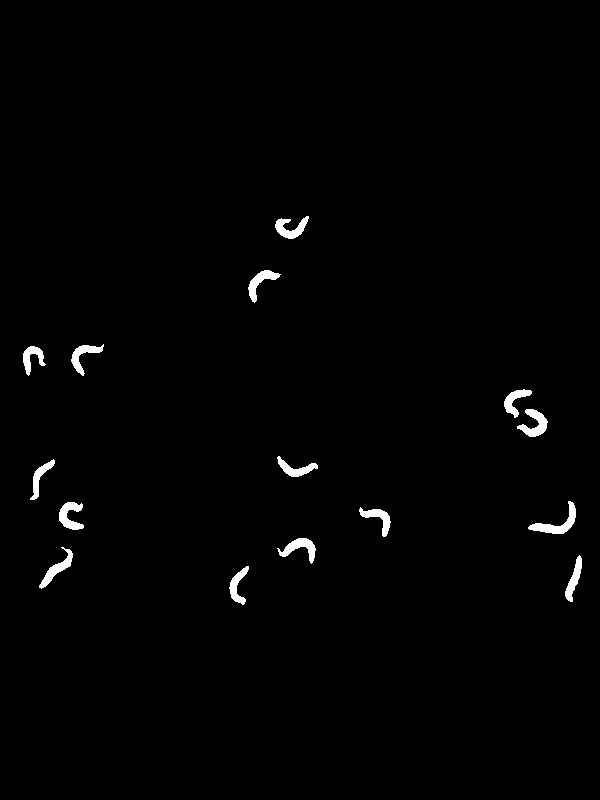

Supplement: S1 File — CellProfiler project files 1–2 are used to train and create worm models. CellProfiler project file 3 is used to score focal and competitor worms in image pairs and output scores as .csv files. (ZIP) [file pone.0201507.s005.zip › File S3/CP_outputs/worm_model_training_output/Assay_12_worm_model_trianing_output/BF_12_1_C05_SingleWorms.png]

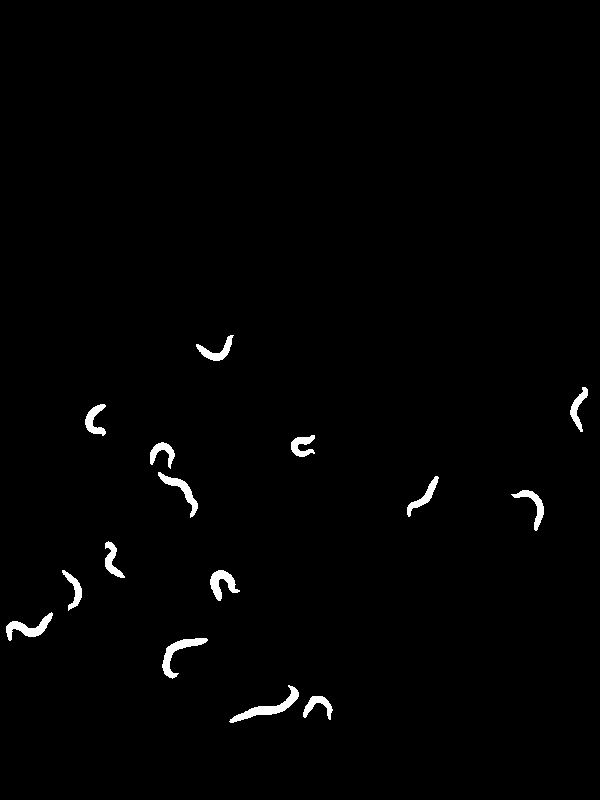

Supplement: S1 File — CellProfiler project files 1–2 are used to train and create worm models. CellProfiler project file 3 is used to score focal and competitor worms in image pairs and output scores as .csv files. (ZIP) [file pone.0201507.s005.zip › File S3/CP_outputs/worm_model_training_output/Assay_12_worm_model_trianing_output/BF_12_1_D02_SingleWorms.png]
